# Supplementary material for: Winter diet of Burrowing Owls in the Llano La Soledad, Galeana, Nuevo León, México
Source: PeerJ. 2022 Apr 27;10:e13324. doi: 10.7717/peerj.13324 (PMC9056000; doi:10.7717/peerj.13324)
Supplement: Supplemental Information 2 [file peerj-10-13324-s002.docx]

Supplementary data. Abbreviations.

| Abbreviation | Definition |
| --- | --- |
| km | Kilometers |
| m | Meters |
| mm | Millimeters |
| FO% | Frequency of occurrence percentage |
| N% | Numerical percentage |
| W% | Weight percentage |
| IRI | Index of relative importance |
| IRI% | Percent IRI |
| CIs | 95% confidence intervals |
| FT | Smith’s measure |
| Ro | Index of Horn |
| ϕc | Cramer’s phi coefficient |
